# Supplementary material for: Mortality rate-dependent variations in antenatal corticosteroid-associated outcomes in very low birth weight infants with 23-34 weeks of gestation: A nationwide cohort study
Source: PLoS One. 2020 Oct 5;15(10):e0240168. doi: 10.1371/journal.pone.0240168 (PMC7535030; doi:10.1371/journal.pone.0240168)
Supplement: Attachment — Submitted filename: Response to reviewer.docx [file pone.0240168.s001.docx]

**Response to reviewer**

Reviewer #1: This article by Park et al., on Mortality rate-dependent variations in antenatal corticosteroid-associated outcomes in very low birth weight infants with 23-34 weeks of gestation: A nationwide cohort study is an important topic with regard to use of antenatal steroids and assess why their effect is different at different GA based on mortality.

Overall, the paper is well written and is clear.

Introduction: provides sufficient background information.

Methods: The data is from 67 neonatal units from KNN registry. Rest of the methodology section is clear.

Results:
Authors have indicated ACS use was 81% in group1 for GA 23-24wk compared to group 2 was 72%.
Mortality was significantly higher for 23-24 wk. in-group 2 compared to group 1 and rest of the GA the mortality rates remained high in-group 2.

The incidence of IVH reduced in 23-24 wk., reduced PVL at 25-26 wk., decreased RDS, early onset sepsis at 29-34 wk. and increased NEC at 23-26 wk. in-group 1 with ACS use.
Symptomatic PDA at 25-26 week, RDS and sepsis at 29-34 wk. was higher in-group 2 with ACS use.
BPD/ROP rates were not different between the groups.

They have not given details of ACS with regard to doses; partial or complete between the groups on infants who received ACS

Discussion:
Authors have quoted important studies showing benefits of ACS use on mortality and morbidities in infants born at 22-34 wk. gestation.

With improved IVH, RDS, PVL, early onset sepsis in Group 1 with mortality <50% with ACS use.

Authors make a good point that with ACS use, some of the morbidities are different when compared based on mortality rate for each gestational age category.

Most morbidities were much improved in infants in group 1 and some morbidities were better (NEC) in group 2 with ACS use

They mentioned in the method section that they were unable to group them based on the neonatal units because of wide institutional variation in the mortality rate in these infants(23-24 wk). Their conclusion that the beneficial effects of ACS use differ not only GA but also by quality of perinatal and NICU care. The gestational age difference with regard to ACS use, with regard to mortality (<50% and > 50%) is shown with their data but their conclusion on ACS use differ on quality of perinatal and NICU care needs more explanation and discussion as it is mentioned they provided active resuscitation to all infants born at 23-24 wks.

Authors are studying the effect of ACS with regard to mortality, it was important to have the information regarding single/multiple, or 1 or 2 doses of ACS, whether that explained some of the difference seen in this study between the groups at different GA.

(Single/multiple or 1 or 2 doses of ACS)

🡪 Thank you for your comment, and we fully agree with your comment that the effects of single/multiple or 1 or 2 doses of ACS on mortality should be further explained.

Just as the reviewer has pointed out, Cochrane systemic review of 2017 (Roberts D, et al. PMID: 28321847) states that “further information is also required concerning the optimal dose-to delivery interval, and the optimal corticosteroid to use” in regards to “antenatal corticosteroids for accelerating fetal lung maturation for women at risk of preterm birth”.

It is rightly so that single/multiple or 1 or 2 doses of ACS and it effect on mortality should have been further dissected, but, the data in our study lacked such details. Therefore, we have added this limitation to the discussion and also explained it in the definition section:” ACS treatment was defined as the administration of any corticosteroid to the mother at any time before delivery to accelerate fetal lung maturity.” (Page 6, Line 117-119)(Page 14, line 18-19)

(Further explanation on difference on quality of perinatal and NICU care)

🡪 We thank the reviewer for the insightful comment.

As the reviewer has pointed out, it is mentioned in the method section that “only VLBWIs actively resuscitated in the delivery room, and admitted to the NICU” were included in this study and those that did not received active resuscitation were excluded. Our intention was to take our included subjects and further compare to stress that ACS use itself is not the only factor affecting the outcome but, in fact, the quality of clinical practices such as well-experienced and skillful neonatologist, better early admission care, and prevention of complication can be of greater influence in increasing survival in 23-24 wk.

We have added “Taken together, these results suggest that improved quality of perinatal and NICU care such as well-experienced and skillful neonatologist, better early admission care, and prevention of complication improved mortality of peri-viable infants at 23-24 weeks gestation. Therefore, higher quality of clinical care can be a prerequisite to the beneficial effects associated with ACS use.” in the discussion section of revised manuscript. (Page 14, line 10-14)

Please explain what do they mean when they say “the potential bias of over-represented women admitted in advanced labor in the without ACS use group was not adjusted for in this study”

(Bias)

🡪 We thank reviewer’s thoughtful comments.

We thank the reviewer’s thoughtful comments. The statement “ the potential bias of over-represented women admitted in advanced labor in the without ACS use group was not adjusted for in this study” was included to point out the bias of hospitals that might have had worse outcome due to greater proportion of mothers in immediate labor who did not have enough time to administer ACS. As the reviewer pointed out, this statement is highly prone to confusion and is not absolutely crucial to point out; therefore, we have removed the statement in revised manuscript.(Page 14, line 19)

Thank you.

We appreciate the thoroughness with which the reviewer examined our paper, and hope that it is now suitable for publication in the prestigious journal “PLOS ONE*.*”

We look forward to hearing from you soon.

Sincerely,

Won Soon Park, MD. PhD.
